# QT Interval Dispersion 36 Years On: Why the Concept Failed and What Replaced It

**DOI:** 10.15190/d.2026.12

**Published:** 2026-09-07

**Authors:** Fernando Rodríguez González, Marianela Ballesteros Hernández, Raimundo Carmona-Puerta, Elizabeth Lorenzo-Martínez, Ionuț Donoiu, Elibet Chávez González

**Affiliations:** ^1^Department of Electrophysiology and Cardiac Pacing, Hospital Universitario Cardiocentro “Ernesto Guevara”, Villa Clara, Cuba; ^2^Universidad de Ciencias Médicas de Villa Clara, Villa Clara, Cuba; ^3^Universidad Católica del Cibao, La Vega, Dominican Republic; ^4^Department of Cardiology, University of Medicine and Pharmacy of Craiova, Craiova, Romania

**Keywords:** QT dispersion, ventricular repolarization, sudden cardiac death, risk stratification, vectorcardiography, electrocardiography.

## Abstract

In 1990, the difference between the longest and the shortest QT interval measured across the twelve leads of the standard electrocardiogram was proposed as a simple and inexpensive index of the spatial heterogeneity of ventricular repolarization. QT dispersion was adopted rapidly and applied to almost every cardiac and to many non-cardiac conditions and was described by its proponents as a potential Holy Grail for the prevention of sudden cardiac death. Within a decade, however, disappointing results from large studies and a series of methodological and conceptual objections had led others to characterise it as the greatest fallacy in electrocardiography of the 1990s. This narrative review reappraises QT dispersion thirty-six years after its description. We examine four separate lines of criticism: the poor reproducibility of T-wave offset determination and the consequent amplification of measurement error in a parameter of small absolute magnitude; the absence of any demonstrated rate dependence, which invalidates the widespread practice of heart-rate correction; the competing local and vectorial explanations of the phenomenon, together with the experimental and clinical evidence that favours projection over regional information; and the inconsistent prognostic performance of the parameter across major cohort studies. We further argue that the failure of QT dispersion is not a failure of the underlying premise that repolarization heterogeneity is arrhythmogenic, but of the specific surface measurement chosen to capture it, and we summarise the contemporary non-invasive markers that have since been developed for the same purpose. We conclude that QT dispersion should not be interpreted as a measure of the dispersion of repolarization duration, and that its residual association with clinical risk is more plausibly explained by projection effects and measurement inaccuracy than by regional electrophysiological information. QT dispersion is still reported without acknowledgement of these objections. Setting them out together with the markers that have replaced the parameter should help readers judge what it can and cannot support.

## SUMMARY

*1. Introduction*

*2. Methods*

*3. Measurement of QT dispersion*

- *3.1. Determination of the end of the T wave*

- *3.2. Reproducibility of manual and automated measurement*

- *3.3. The onset of the QT interval*

- *3.4. Reference values*

*4. Heart rate and QT dispersion*

*5. The phenomenological basis of QT dispersion*

- *5.1. The local theory*

- *5.2. The global, projection or vector theory*

- *5.3. Evidence from lead geometry and from derived electrocardiograms*

- *5.4. Non-dipolar components of the T wave*

- *5.5. Is QT dispersion actually dispersed?*

- *5.6. Evidence from direct epicardial measurement*

*6. Clinical utility*

*7. Beyond QT dispersion: contemporary markers of repolarization heterogeneity*

- *7.1. The Tpeak-Tend interval*

- *7.2. The spatial QRS-T angle*

- *7.3. Principal component analysis of the T wave*

- *7.4. Electrocardiographic imaging*

- *7.5. Artificial intelligence applied to the electrocardiogram*

- *7.6. What the failure of QT dispersion teaches*

*8. Final considerations*

*9. Limitations of this review*

*10. Conclusions*

## 1. Introduction

In 1990, the group led by Campbell revived the proposal that the regional heterogeneity of ventricular repolarization could be derived from the surface electrocardiogram (ECG) by measuring inter-lead differences in the duration of the QT interval. The difference between the longest and the shortest QT interval recorded across the twelve conventional leads, termed QT dispersion (QTd), was suggested as an index of the spatial dispersion of ventricular recovery times^1^. The underlying hypothesis was that, because the ECG leads occupy different positions relative to the chest, each records the electrical activity of a distinct myocardial region; QTd would therefore constitute a measure of repolarization heterogeneity^2^. The proposal was received enthusiastically. Its methodological simplicity and negligible cost made the prospect of obtaining regional electrophysiological information non-invasively, from a standard twelve-lead recording, extremely attractive^3^. The literature was subsequently flooded with reports of QTd in virtually every cardiac syndrome and in a large number of non-cardiac conditions. Doubts about the validity of the concept, and about the reliability of its measurement, were nevertheless expressed repeatedly and from an early stage^4^. The distance between expectation and evidence is captured by the language used on either side of the debate. QTd was described by its advocates as a potential Holy Grail for the prevention of sudden cardiac death (SCD)^5^; within a decade, following a series of disappointing results from large studies, it had been labelled the greatest fallacy in electrocardiography of the 1990s^6^. By 2002 the question posed in the specialist literature was no longer whether QTd was useful, but why it had died^7^. More than three decades later, QTd still appears in the literature, often without acknowledgement of the objections raised against it. This narrative review sets out the evidence for and against the parameter thirty-six years after its description, and places it alongside the non-invasive markers of repolarization heterogeneity developed since.

## 2. Methods

This is a narrative review. PubMed/MEDLINE, Embase and Web of Science were searched from inception to May 2026 using combinations of the terms “QT dispersion”, “dispersion of ventricular repolarization”, “interlead heterogeneity”, “T-wave offset”, “T-loop morphology”, “non-dipolar components” and “sudden cardiac death”. Reference lists of the retrieved articles and of relevant book chapters were screened manually for additional sources. Articles in English and Spanish were considered. Because the aim was to reconstruct and appraise a scientific controversy rather than to pool effect estimates, no formal quality-scoring instrument was applied and no quantitative synthesis was undertaken; priority was given to the primary methodological, experimental and large-cohort literature, and to publications that articulate the opposing positions in the debate.

## 3. Measurement of QT dispersion

### 3.1. Determination of the end of the T wave

The principal difficulty in measuring the QT interval is the identification of the end of the T wave, and this is also the principal source of error in the determination of QTd^8^. A second problem is that QTd is a small quantity relative to the QT interval itself. A modest absolute error in the measurement of the QT interval is therefore magnified when the difference between two such measurements is taken. Increasing paper speed may paradoxically render the T-wave offset more ambiguous when it is not accompanied by a corresponding increase in gain^9^.

**Figure 1:**
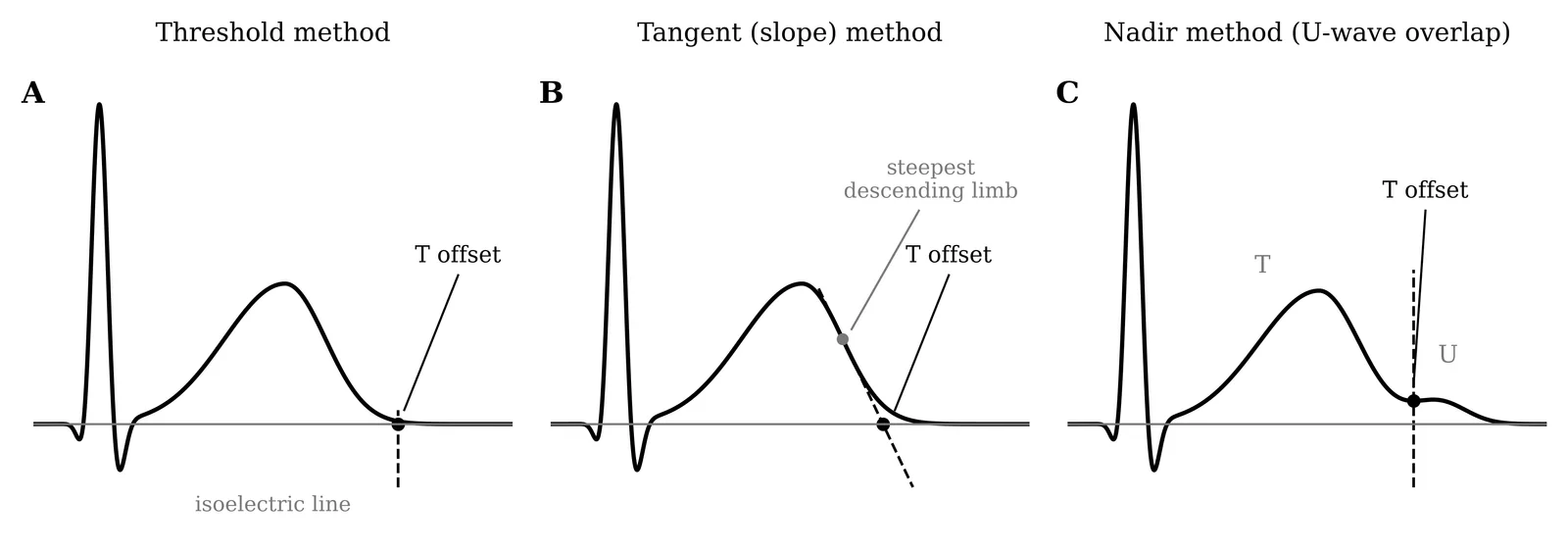
Schematic representation of the three methods most frequently used to determine the end of the T wave (**A**) Threshold method: the T-wave offset is taken at the intersection of the descending limb with the isoelectric line. (**B**) Tangent (slope) method: a line drawn along the steepest portion of the descending limb is extrapolated to the isoelectric line. (**C**) Nadir method applied when a U wave overlaps the terminal portion of the T wave: the offset is taken at the trough between the two waves. The three methods do not yield identical values in the same complex, and the choice of method is one determinant of the reported magnitude of QT dispersion.

Although low T-wave amplitude is the dominant source of error, superimposition of U waves or of P waves may also introduce bias. Several algorithms for determining the end of the T wave according to its morphology have been proposed^9^. The threshold method takes the point at which the descending limb of the T wave meets the isoelectric line. Where that intersection cannot be identified precisely, the slope or tangent method is used: a line is drawn along the steepest portion of the descending limb and extrapolated to the isoelectric line. When a U wave overlaps the terminal T wave, the nadir method is preferred, in which a vertical line is dropped from the trough between the two waves (**Figure 1**).

### 3.2. Reproducibility of manual and automated measurement

Several studies have documented high inter-observer variability for manual measurements. The relative inter-observer error for QTd has been estimated at 25–40%, against less than 6% for the QT interval itself^2^. Other reports have attributed a considerably smaller error to the measurement method. The use of digital callipers, signal amplification, paper speed, the presence of noise and the use of a digitising tablet all influence the precision of the result^10^.

In a further analysis, inter-observer variation in locating the end of the T wave was 30.6 ms, against 6.5 ms for the onset of the QRS complex. It was expected that digital systems would improve the homogeneity of measurement substantially. When nineteen automated systems were compared, however, the difference for the T-wave offset was again of the order of 30 ms, against approximately 6 ms for QRS onset, and the accuracy of QT interval determination was of the same order for manual and automated modalities^11^. T-wave morphology contributes substantially to this poor reproducibility; the complexity and wide variability of repolarization patterns plausibly explain the broad spectrum of values reported in the literature.

A computational approach based on convolutional neural networks trained on expert-annotated recordings has reduced the discrepancy between manual and automatic measurement, although its authors recommend manual confirmation where T-wave morphology is complex^12^. That method was developed and validated for the QT interval in drug safety assessment, not for QTd, and it inherits the same difficulty with atypical T waves. Accurate evaluation of QTd requires twelve leads recorded simultaneously, to remove dynamic QT changes arising from beat-to-beat variation in heart rate.

### 3.3. The onset of the QT interval

Dispersion of the QRS onset, although far smaller in magnitude than dispersion of the T-wave offset, also contributes to QTd. In manual measurement, the onset of the Q wave is determined independently in each lead. Computational algorithms may either determine the onset separately in each lead or apply a single common fiducial point across all leads. These differing conventions account in part for the variability of reported QTd values^11^.

### 3.4. Reference values

A review of the QTd literature reveals a wide spread of reported values with no discernible convergence towards accepted reference limits. Some studies report an upper limit of normal of 65 ms in healthy subjects, with no significant difference between sexes or across age groups^13^. A meta-analytic summary found a mean normal value below 40 ms in thirteen studies and above that figure in eight others^3^. A review of the value of the surface ECG in predicting SCD placed the normal range at 40–50 ms with a maximum of 65 ms^13^. Values above 65 ms have been said to identify a high risk of ventricular arrhythmias and SCD, although it is likely that only markedly abnormal values, above approximately 100 ms and therefore outside the range of measurement error, could have practical value as an indicator of abnormal repolarization^3^.

A cut-off of 50 ms yields a sensitivity of 92% with a specificity of 43%, whereas the proposed upper permissible limit of 80 ms yields a specificity of 89% with a sensitivity of 67%^3^. These figures are summarised in **Table 1**. No standardised protocol for determining the parameter exists, and reported reference values show no trend towards agreement.

**Table 1:** Reported reference values and proposed cut-off points for QT dispersion ECG, electrocardiogram; SCD, sudden cardiac death

Source / context	Reported value	Comment
Healthy subjects^13^	Upper limit of normal 65 ms	No significant difference by sex or age
Meta-analytic summary^3^	Mean normal value <40 ms in 13 studies; >40 ms in 8 studies	No convergence towards an agreed reference limit
Review of the surface ECG in SCD prediction^13^	Normal range 40–50 ms; maximum 65 ms	Values >65 ms proposed as indicating high arrhythmic risk
Threshold outside the range of measurement error^3^	>100 ms	Only markedly abnormal values are likely to have practical value
Cut-off point 50 ms^3^	Sensitivity 92%; specificity 43%	High sensitivity obtained at the cost of specificity
Proposed upper permissible limit 80 ms^3^	Sensitivity 67%; specificity 89%	The reciprocal trade-off; no consensus cut-off exists

Moreover, most investigators have reported extensive overlap of values between healthy individuals and patient groups. All proposed upper limits of normal should therefore be regarded as unreliable. The number of leads analysed, the convention adopted for QRS onset, T-wave morphology, the method used to identify the T-wave offset, sweep speed and recorder gain all affect the values obtained.

## 4. Heart rate and QT dispersion

One of the persistent uncertainties concerns whether QTd should be corrected for heart rate. Most early clinical studies applied a heart-rate correction algorithm to QTd, generally based on Bazett's formula, on the assumption that the dispersion of ventricular repolarization possessed a rate dependence similar to that of its duration^3^.

The physiological basis of the adaptation of the QT interval to heart rate has been well established, and several algorithms have accordingly been recommended for clinical correction of QT interval duration^14^. The dispersion of ventricular repolarization, by contrast, was shown to be independent of heart rate in an experimental study using multiple monophasic action potentials recorded from isolated rabbit hearts paced at different rates. This independence was subsequently confirmed in healthy subjects and in patients with relatively mild heart disease^15^.

Corrected QTd was nevertheless in widespread use before the rate independence of repolarization dispersion was demonstrated and was even proposed as a superior risk stratifier^16^. In our interpretation, heart-rate correction introduced an unnecessary variable into the measurement; this inference has not been tested directly. Heart rate is itself a well-established risk stratifier^17^ and building it into a composite index adds an independent risk marker whose predictive contribution is then attributed to the dispersion measurement.

In principle, correcting any measure of repolarization for heart rate is justified only where that measure has been shown to depend on heart rate. Ample evidence establishes that this condition is met for the duration of the QT interval; there is no comparable confirmation for QTd. The use of rate-corrected QTd values without analysis of the original uncorrected data is therefore inappropriate, both because there is no evidence that QTd requires the same form of correction as QT interval duration, and because the difference between measured and corrected values may conceal an underlying influence of heart rate that could be the sole explanation for results reported over past decades.

## 5. The phenomenological basis of QT dispersion

Two principal theories have been advanced to explain the origin of QTd.

### 5.1. The local theory

The local theory rests on regional dispersion of repolarization, which would produce differences in the duration of transmural action potentials or between different segments of the ventricular myocardium^14^. On this account, because the ECG leads are arranged so as to record cardiac electrical activity from twelve distinct positions, they may register QT intervals of differing duration according to the myocardial region explored. Ventricular areas with slower repolarization would give rise to longer QT intervals, and the converse would hold where the region explored by a given lead exhibited shorter action potentials. These differences in duration would constitute the phenomenon of QTd.

### 5.2. The global, projection or vector theory

The second explanation is the global, projection or vector theory. It rests on the principles of vector electrocardiography and consists of the projection of the ventricular repolarization vector onto the axis system of the frontal and horizontal plane leads^18^.

Applying the elementary principles of coordinate representation in two dimensions, and without disregarding the magnitude of the vector, the more nearly parallel its direction is to a given coordinate axis, the more completely it will project onto that axis. For an electrocardiographic lead, this yields a QRS complex and a T wave inscribed over their full extent, with no portion of the wave failing to appear. At the opposite extreme, a vector oriented perpendicular to a coordinate axis cannot project onto it at all; the electrocardiographic equivalent is the absence of any inscription of that vector in the corresponding lead.

**Figure 2:**
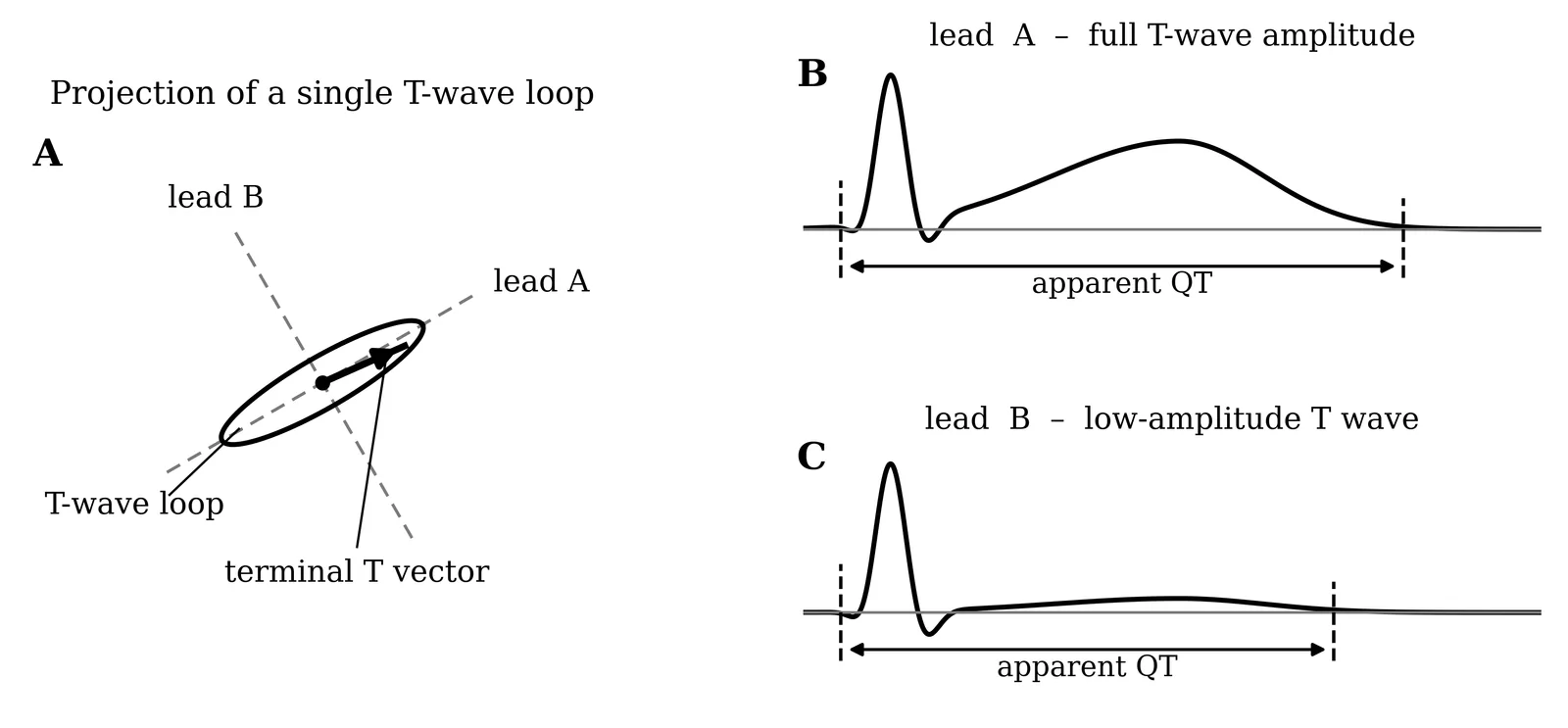
The projection mechanism as an explanation of inter-lead differences in QT interval duration (**A**) A narrow, elongated T-wave loop and two lead axes: lead A is oriented nearly parallel to the terminal repolarization vector, lead B nearly perpendicular to it. (**B**) In lead A the loop projects almost completely and the T wave is inscribed at full amplitude, so the terminal portion of repolarization is detected and the apparent QT interval is long. (**C**) In lead B the projection is small, the terminal portion of the T wave falls below the threshold of visual or algorithmic detection, and the apparent QT interval is correspondingly shorter. The difference between the two leads arises entirely from the geometry of projection, without any difference in regional repolarization duration.

Because the components of the QT interval in any lead are inscribed as the electrocardiograph detects the multiple vectors that compose it, longer or shorter QT intervals will arise where the projections of a common vector are more nearly parallel or more nearly perpendicular in particular leads during particular fractions of the cycle. This tends to occur chiefly at the beginning and the end of ventricular depolarization and repolarization, where the potentials generated are of smaller magnitude and are therefore more susceptible to escaping inscription. The result is a portion of the wave of very low amplitude, or an apparently isoelectric segment, detectable only at high magnification (**Figure 2**).

### 5.3. Evidence from lead geometry and from derived electrocardiograms

Numerous clinical investigations support the vectorial account. Kors and colleagues showed that the difference between the maximum QT interval and the QT measured in leads whose axis ran parallel to the terminal repolarization vector was null or of very little magnitude. Lower QT values were recorded in leads oriented perpendicular to that vector. Where the angle between the terminal vector and the lead axis was less than 30 degrees, differences were minimal, though slightly smaller than for vectors exactly aligned with the lead axis^18^.

A close relationship between the spatial amplitude of the T-wave loop, the T wave and the QT interval has been demonstrated^19^. Elongated, narrow loops correspond to lower dispersion values, whereas loops of reduced amplitude and greater width, approaching a circular configuration, correspond to higher dispersion values. Both the amplitude and the duration of the vector loop are therefore determinants of QT interval duration and, consequently, of QTd.

This has an important implication. The vector loop, as a single image of repolarization, cannot itself generate dispersion. Any projection onto a lead axis not parallel to it loses part of the information, and the variation of that projection across leads may be no more than a measure of what has been lost. Where the vector runs perpendicular to an axis its projection becomes null, as though the electrical activity had disappeared^19^.

Macfarlane showed that a standard twelve-lead ECG and one derived from the Frank orthogonal leads differed in QTd by less than 2 ms^11^. Lee and colleagues obtained comparable results in 129 subjects^20^. Derived electrocardiograms contain no local information, being the product of projecting the vector loop onto each lead axis. Divergent results between standard and derived recordings would have supported the local theory; the similarity of the values obtained supports the vectorial account.

Somberg has dissented from this interpretation, arguing that myocardial activity does not consist of a single dipole but of a dipole whose magnitude, direction and polarity change continuously under the influence of local and regional differences. On his account, spatial vector recordings do contain regional information precisely because the pattern of these instantaneous changes is shaped by regional differences in repolarization, and a derived electrocardiogram differs from the original no more than the error of the conversion algorithm; the derivation therefore neither distorts nor removes the regional information contained in the signal^21^.

### 5.4. Non-dipolar components of the T wave

A necessary, though not sufficient, condition for the validity of the QTd concept is the presence of non-dipolar components in the body-surface ECG during ventricular repolarization, and that these components be of sufficient magnitude to show a significant association with QTd^22^.

The publication by Malik and colleagues in 2000 was the first to document substantial non-dipolar components in the T wave of standard twelve-lead recordings from healthy subjects, from patients with hypertrophic and dilated cardiomyopathy, and from survivors of acute myocardial infarction. QTd did not, however, correlate significantly with these components, apart from a marginal correlation in patients with hypertrophic cardiomyopathy, leading the authors to conclude that QTd bears no relationship to the non-dipolar content of the T wave^22^.

Rautaharju subsequently confirmed these findings in a much larger sample, demonstrating non-dipolar components in the T waves of twelve-lead recordings with a mean amplitude of 11 µV, exceeding 18 µV in 5% of subjects. These values fall below the limits of visual resolution and are smaller than the amplitude thresholds used by many computer algorithms to define the end of the T wave. In the same analysis, non-dipolar voltage alone accounted for only a small fraction of the variance in QTd^7^.

### 5.5. Is QT dispersion actually dispersed?

The word dispersion derives from the Latin dispergere, to scatter, and implies separation and movement in different directions without order or regularity. It is therefore legitimate to ask whether the distribution of QT intervals across the leads is truly dispersed, or whether some degree of regularity exists in the distribution between adjacent leads. The rationale for the question is that irregularity of the functional refractory period, or of local repolarization time, over relatively short distances is what increases the propensity to triggered activity and re-entry. Localised myocardial dispersion of this kind should therefore produce QTd between adjacent leads.

A test of the local theory along these lines would be the demonstration of extreme dispersion between contiguous leads. In the analysis reported by Rautaharju, the highest and lowest values in adjacent horizontal-plane leads occurred in only 27% of contiguous pairs, most frequently between V1 and V2, and the higher frequency in the right precordial leads is attributable in part to the wide angle between them^7^. The distribution is thus not that of a scattered quantity, but one shaped by lead geometry.

### 5.6. Evidence from direct epicardial measurement

A further approach is to compare QT intervals measured at the body surface with direct measurements of epicardial repolarization. To this end, a canine heart was perfused by the Langendorff technique and suspended in a torso-shaped electrolytic tank filled with a sucrose and saline mixture. An epicardial array of 64 silver wire electrodes was placed on the heart, which was paced at a fixed cycle length of 350 ms, and a 12 × 16 array on the surface of the tank provided 192 torso-surface electrocardiograms. Recordings of 3 to 5 seconds were obtained at different temperatures and from different pacing sites. The correlation between the dispersion of repolarization recorded at the epicardium and QTd measured at the torso surface was low^23^.

QTd therefore does not reflect the dispersion of repolarization duration even when compared against direct myocardial measurement. The two theories nevertheless continue to divide the field, and the clinical literature contains contradictory results. The measurement also disregards the T wave itself, yielding only the difference between the maximum and minimum QT interval and discarding the container of ventricular repolarization. Other authors have proposed the voltage domain as a more appropriate framework for studying spatial dispersion of repolarization^24^.

## 6. Clinical utility

QTd has been presented as both a diagnostic and a prognostic tool, predictive of overall and of arrhythmic mortality. The principal cohort studies are summarised in **Table 2**.^16,25-33^ In the Strong Heart Study, which enrolled 1,839 American Indians from seven communities, QTd proved a significant predictor of all-cause and cardiovascular mortality^25^. Comparable results were obtained in the Rotterdam Study, in which more than 6,000 participants aged over 55 years were followed clinically with serial electrocardiographic recordings over a mean of approximately four years; a rate-corrected QTd above 60 ms was a strong and independent predictor of cardiac mortality^16^.

**Table 2:** Principal clinical studies of QT dispersion as a prognostic marker SCD, sudden cardiac death; UK-HEART, United Kingdom Heart Failure Evaluation and Assessment of Risk Trial.

Study	Population	n	Follow-up	Main finding
Strong Heart Study^25^	American Indians from seven communities	1,839	Mean 3.7 ± 0.9 years	QT dispersion an independent predictor of all-cause and cardiovascular mortality
Rotterdam Study^16^	Community-dwelling subjects aged >55 years	>6,000	Mean ≈ 4 years	Rate-corrected QT dispersion >60 ms an independent predictor of cardiac mortality
UK-HEART^26^	Ambulatory mild-to-moderate congestive heart failure	>500	Mean 471 days	QT dispersion not an independent marker of all-cause mortality, SCD or disease progression
Yi et al.^27^	Hypertrophic cardiomyopathy	156	Not applicable (cross-sectional study)	QT dispersion unrelated to established SCD risk factors; weak correlation with wall thickness
Chávez-González et al.^32^	ST-elevation myocardial infarction	667	In-hospital	Corrected QT dispersion >60 ms independently associated with ventricular arrhythmias, but with a smaller effect size than QRS duration, QRS dispersion or extent of ST-segment elevation
Zabel et al.^33^	Survivors of myocardial infarction (prospective)	280	32 ± 10 months	None of 26 indices of repolarization dispersion predicted arrhythmic events or mortality

A substudy of the United Kingdom Heart Failure Evaluation and Assessment of Risk Trial (UK-HEART), comprising more than 500 outpatients with mild-to-moderate congestive heart failure followed for a mean of 471 days, found by contrast that QTd was not an independent marker of all-cause mortality, of SCD or of disease progression^26^.

In an evaluation of QTd against established risk factors for SCD in hypertrophic cardiomyopathy, 156 patients were assessed. QTd was similar irrespective of the number of recognised risk factors present, including family history of sudden death, non-sustained ventricular tachycardia, exertional angina and functional class, and only a weak correlation with ventricular wall thickness was observed^27^.

Numerous studies have examined the value of QTd in predicting ventricular arrhythmias or other adverse events across a range of cardiac diseases, and here too the results are inconsistent. Although most report significantly higher QTd in patients with arrhythmias, the distributions overlap extensively. Several studies, predominantly retrospective, found that patients with acute or chronic myocardial infarction who developed ventricular arrhythmias exhibited significantly greater QTd than those who did not^28-31^.

A more recent observational study of 667 patients with ST-elevation myocardial infarction shows both the persistence of the association and the difficulty of interpreting it. Corrected QTd above 60 ms was independently associated with ventricular arrhythmias, but with the smallest effect size among the significant electrocardiographic predictors (odds ratio 1.81, 95% confidence interval 1.05 to 3.12), and was outperformed by QRS duration, by the extent of ST-segment elevation and by QRS dispersion; beyond 48 hours the main predictor was QRS dispersion, not QT dispersion^32^. Two features of that analysis bear on the argument above. The dispersion values were rate-corrected, a practice for which no justification has been established, and they were measured manually at 25 mm/s and 10 mm/mV, the conditions under which error in locating the T-wave offset is greatest.

The first prospective study in survivors of myocardial infarction showed that none of the 26 indices of ventricular repolarization dispersion evaluated had predictive value for adverse outcomes in 280 consecutive patients followed for 32 ± 10 months^33^.

## 7. Beyond QT dispersion: contemporary markers of repolarization heterogeneity

The decline of QTd did not end interest in the underlying question. Heterogeneity of ventricular repolarization remains an established arrhythmogenic substrate, and the search for a non-invasive index that captures it has continued. Each of the more recent approaches addresses one of the defects identified above: reliance on a single poorly reproducible fiducial point, the discarding of the T wave, or the confusion of projection with regional information. **Table 3** presents the comparison.

**Table 3:** Contemporary non-invasive markers of ventricular repolarization heterogeneity ECG, electrocardiogram; SCD, sudden cardiac death; Tpe/QT, ratio of the Tpeak-Tend interval to the QT interval.

Marker	What it measures	Principal supporting evidence	Main limitation
Tpeak–Tend interval and Tpe/QT ratio^34-36^	Transmural dispersion of repolarization	Meta-analysis of 33 studies (155,856 patients); independent association with SCD in coronary artery disease	Depends on the same poorly reproducible T-wave offset; lead selection and rate correction unresolved
Spatial and frontal QRS–T angle^37^	Discordance between the depolarization and repolarization vectors	Meta-analysis of 22 studies (164,171 subjects) for all-cause and cardiac death	Requires vectorcardiographic derivation; a broad prognostic rather than a specific arrhythmic marker
Principal component analysis of the T wave^38^	Dipolar and non-dipolar content and complexity of repolarization	Predicted cardiovascular mortality independently of QT interval and QT dispersion	Requires digital signal processing; not reported by standard electrocardiographs
Electrocardiographic imaging^39,40^	Epicardial activation-recovery intervals and regional repolarization gradients	Validated against direct epicardial mapping	Cost, requirement for tomographic geometry, limited availability
Artificial intelligence applied to the ECG^41,42^	Data-driven features not accessible to visual inspection	Deep learning models for SCD risk and for cardiovascular mortality	Opacity of derived features; heterogeneous datasets; limited prospective validation

### 7.1. The Tpeak-Tend interval

The interval from the peak to the end of the T wave has been proposed as a marker of transmural dispersion of repolarization. A systematic review and meta-analysis of 33 observational studies comprising 155,856 patients found that prolongation of the interval predicted arrhythmic and mortality outcomes, with the association strongest in Brugada syndrome^34^. A prolonged Tpeak–Tend interval has also been independently associated with SCD in patients with coronary artery disease, including among those in whom the corrected QT interval was normal or uninterpretable because of intraventricular conduction delay^35^.

The parameter is not free of the criticism directed at QTd. It depends on the same determination of the T-wave offset; the lead in which it should be measured, the appropriate rate correction and the definition of the T-wave peak in biphasic or notched morphologies all remain unsettled, and a critical appraisal by the e-Rhythm Study Group of the European Heart Rhythm Association has questioned whether the published evidence supports its clinical use^36^. The lesson of QTd applies here: an index built on a poorly reproducible fiducial point carries that imprecision into every subsequent analysis.

### 7.2. The spatial QRS-T angle

The angle between the mean or peak QRS vector and the T vector quantifies the discordance between the sequences of depolarization and repolarization. Because it is derived from the geometry of the loops rather than from a single time point, it is less dependent on the identification of wave offsets. A meta-analysis of 22 studies enrolling 164,171 individuals found that a wide spatial QRS-T angle was associated with all-cause death and with cardiac death, and that a wide frontal angle also predicted all-cause death^37^. The parameter has the advantage of a coherent electrophysiological rationale and of reasonable reproducibility, but it is a broad prognostic marker rather than a specific index of arrhythmic risk.

### 7.3. Principal component analysis of the T wave

Principal component analysis decomposes the repolarization signal into its dipolar and non-dipolar constituents and yields indices of T-wave complexity, most commonly the ratio of the second to the first eigenvalue. This approach addresses directly the objection that QTd discards the T wave: rather than reducing repolarization to the difference between two time points, it characterises the morphology of the wave itself. In the Strong Heart Study cohort, T-wave complexity derived in this way predicted cardiovascular mortality independently of the QT interval and of QTd^38^. Its limitation is practical rather than conceptual, in that it requires digital signal processing and is not reported by standard electrocardiographs.

### 7.4. Electrocardiographic imaging

Electrocardiographic imaging reconstructs epicardial potentials, electrograms and activation-recovery intervals from several hundred body-surface electrodes combined with patient-specific heart–torso geometry derived from computed tomography or magnetic resonance imaging^39^. Because it solves the inverse problem explicitly rather than relying on the projections captured by twelve leads, it offers genuinely regional information of the kind that QTd was mistakenly assumed to provide. Validation against direct epicardial mapping has shown that regional repolarization gradients are reconstructed with reasonable accuracy^40^. Cost, the requirement for tomographic imaging and limited availability currently restrict the technique to specialist centres and to research.

### 7.5. Artificial intelligence applied to the electrocardiogram

Deep learning models trained on the digital twelve-lead signal identify features that are not accessible to visual inspection and that do not correspond to any conventional interval or amplitude. Models of this type have been developed for the assessment of SCD risk^41^ and for the estimation of general cardiovascular risk and mortality^42^, in several instances outperforming conventional electrocardiographic criteria. Related developments in ambulatory and wearable recording have extended repolarization monitoring beyond the resting recording^43^. The principal reservations concern the opacity of the derived features, the heterogeneity of training datasets and endpoint definitions, and the scarcity of prospective external validation; on present evidence these models should be regarded as promising but not established.

### 7.6. What the failure of QT dispersion teaches

One lesson is methodological. Any index built on the end of the T wave inherits the reproducibility problem that undermined QTd, and the burden of showing that a proposed marker exceeds its own measurement error should be met before large-scale prognostic studies begin. A second is conceptual. A scalar quantity extracted from the surface ECG reflects the projection of a global electrical field and cannot be assumed to carry regional information because the electrodes are spatially separated. The distinction between a global and a local effect must be established, not asserted.

The second lesson extends beyond ventricular repolarization. Dispersion of the P wave, long used to predict atrial fibrillation^44,45^, has been subjected to the same two explanations. In the only study to test both at once, in 153 patients undergoing electrophysiological study with atrial conduction times measured directly, vectorial projection accounted for almost the whole phenomenon, inhomogeneous conduction contributing marginally and chiefly where conduction times were prolonged^46^. Others have argued that P-wave dispersion as conventionally defined corresponds to no real physiological quantity^47^. Dispersion of the QRS complex, built the same way from inter-lead differences, has been reported in association with mechanical desynchrony during permanent right ventricular apical pacing^48^, where altered activation geometry alone explains the inter-lead differences in projected duration. Wherever an index is built from differences between the projections of a single loop onto the lead axes, the interpretation offered here for the T wave is likely to apply.

The electrophysiological substrate is also understood differently today. Repolarization heterogeneity arises at the level of ion channel expression, trafficking and cooperative gating within channel clusters, on spatial scales far below the resolution of any surface measurement^49^. An index derived from twelve chest and limb electrodes could not be expected to capture such organisation. Contemporary reviews of SCD risk prediction assign no independent role to QTd and emphasise multivariable and multimodality approaches ^50,51^.

## 8. Final considerations

Classifying and quantifying regional information from the twelve-lead ECG remains a difficult problem. Despite the initial expectations, it is now clear that QTd has not solved it, since the parameter reflects global abnormality of repolarization rather than localised abnormality.

It is erroneous to assume that the end of the T wave in a given lead is directly related to action potential durations in a corresponding region of the heart. At the end of the cardiac cycle, when the last myocardial cells complete repolarization, the electric field generated by these final active sources spreads throughout the torso and is detected by every electrode placed on the body. At all electrode sites, therefore, the repolarization potentials must have practically the same duration. This is a consequence of elementary electric field theory.

On the surface ECG the potential at an individual electrode cannot be measured; only the potential difference between two conducting electrodes is accessible. If the electrical signal at an electrode falls to zero, electrode potentials equalise. It should be recalled that the precordial electrodes are in effect bipolar, since the central terminal is by no means at zero potential. There is a single end of repolarization, a common endpoint for all electrodes, at which the electric field dissolves and all potential differences disappear.

The very idea of detecting and quantifying only the dispersion of the end of repolarization, that is, the dispersion of complete recovery times, also appears questionable. Action potentials of different duration frequently differ in shape, particularly during phase 3. This phase 3 dispersion, the dispersion of partial recovery times, bears a direct relationship to arrhythmogenesis. It is reflected in T-wave morphology, but it does not contribute to the dispersion of action potential endpoints, and still less to QTd.

We do not question the role of electrophysiological heterogeneity in arrhythmogenesis, nor the evidence that measured QTd may have positive predictive value. We question the interpretation of QTd as a measure of the dispersion of repolarization duration. QTd did not become the clinical tool it was proclaimed to be in the 1990s, and it has not established itself as a prognostic marker of the weight of left ventricular ejection fraction, heart rate variability or heart rate itself.

Although the association between measured QTd and clinical risk is in some settings robust, the mechanism and the significance of that association remain unclear. QTd may arise from underlying heterogeneity of ventricular repolarization, which would be a local effect; but it may equally result from the variable projection of a single repolarization vector across the different leads, which is a projection phenomenon, and from measurement inaccuracy where T-wave amplitude is low and the T-wave offset correspondingly difficult to define. A local effect would imply that surface electrodes record localised myocardial repolarization abnormalities. In a global electric field generated by the heart and represented by the cardiac dipole, however, a local effect would be imperceptible, and QTd measured from surface recordings would be attributable entirely to projection and to measurement error.

## 9. Limitations of this review

This was a narrative review, not preregistered. Study selection was not duplicated, no formal risk-of-bias tool was applied, and the evidence was not pooled. Only publications in English and Spanish were considered. The studies discussed span more than three decades, over which recording equipment, measurement conventions and background therapy all changed, limiting direct comparison of reported values. The markers in Section 7 are treated in outline; a full appraisal of each would require a separate review. Several arguments advanced here are interpretive rather than empirical and are identified as such in the text.

## 10. Conclusions

Thirty-six years after its description, QT dispersion cannot be regarded as a valid measure of the spatial dispersion of ventricular repolarization duration. The parameter lacks a standardised measurement protocol and reference values; its magnitude is dominated by the error inherent in locating the end of the T wave; it has no demonstrated dependence on heart rate, which invalidates the widespread practice of rate correction; it does not correlate with the non-dipolar content of the T wave; and it correlates poorly with directly measured epicardial repolarization dispersion. Its prognostic performance has been inconsistent across major cohorts, and where an association with outcome exists it is more plausibly explained by projection effects and measurement inaccuracy than by regional electrophysiological information.

None of this diminishes the importance of repolarization heterogeneity as an arrhythmogenic substrate. It shows that the surface measurement chosen in 1990 was inadequate to the task. Whether the methods now under development will improve risk stratification for patients at risk of life-threatening arrhythmias remains to be established. The history of QT dispersion suggests the answer will depend less on any reported association with outcome than on whether the index can first be shown to measure what it claims to measure. The same standard should be applied to the data-driven markers now entering the field.

## Author Contributions

Conceptualization, F.R.G. and E.C.G.; writing, original draft preparation, F.R.G. and E.C.G.; writing, review and editing, M.B.H., R.C.P., E.L.M. and I.D.; figure preparation, F.R.G. and I.D.; project administration, E.C.G. All authors have read and agreed to the published version of the manuscript.

## Author ORCID iDs

Rodríguez González 0000-0002-8135-3902; Marianela Ballesteros Hernández 0000-0003-2687-6302; Raimundo Carmona-Puerta 0000-0003-2246-1089; Elizabeth Lorenzo-Martínez 0000-0001-8293-5392; Ionuț Donoiu 0000-0001-6338-2657; Elibet Chávez González 0000-0003-2246-2137.

## Declaration of Generative AI and AI-assisted technologies

During the preparation of this work the authors used a generative AI assistant (Claude, Anthropic) for language editing, figure rendering, restructuring of the manuscript, and preparation of the tables. It was not used for generating any of the scientific ideas, interpretations, analysis, or conclusions. The authors reviewed and verified all AI-assisted output, and take full responsibility for the final content.

## Publisher’s note

All claims expressed in this article are solely those of the authors and do not necessarily represent those of their affiliated organizations, or those of the publisher, the editors and the reviewers. Any product that may be evaluated in this article, or claim that may be made by its manufacturer, is not guaranteed or endorsed by the publisher.
